# Psoas Abscess After Lumbar Facet Joint Injection: Case Report and Literature Review

**DOI:** 10.7759/cureus.9559

**Published:** 2020-08-04

**Authors:** Matheus F. M Ballestero, Vinícius Carneiro, Jose Paulo Luz Lima, Ricardo Santos de Oliveira

**Affiliations:** 1 Department of Medicine, Federal University of São Carlos, Sao Carlos, BRA; 2 Department of Surgery and Anatomy, Clinics Hospital of Ribeirão Preto of the University of São Paulo, University of São Paulo, Ribeirão Preto, BRA; 3 Department of Neurology and Neurosurgery, São Paulo Hospital, São Paulo Hospital, Araraquara, BRA; 4 Division of Neurosurgery, Department of Surgery and Anatomy, Clinics Hospital of Ribeirão Preto of the University of São Paulo, Ribeirão Preto Medical School, University of São Paulo, Ribeirão Preto, BRA; 5 Department of Neurology and Neurosurgery, São Paulo Hospital, Araraquara, BRA

**Keywords:** low back pain, epidural steroid injection, psoas muscle, infection

## Abstract

Low back pain (LBP) is a common condition. It is estimated that 84% of adults will present LBP symptoms at some point in their lives. Rarely, however, is LPB an indication of a serious medical condition, requiring further investigation. The treatment of non-specific LBP is based on non-pharmacological strategies, e.g., non-steroidal anti-inflammatory drugs (NSAID) or skeletal muscle relaxants. The use of epidural steroid injection (ESI) or facet joint injections relieves pain originating from degenerative spine disorders or a disk herniation, thereby providing rapid pain improvement, despite considerable long-term outcomes. Although rare, ESI complications can occur, and infections are infrequently described. This paper describes a rare case of an abscess in the psoas muscle, secondary to facet joint lumbar block. We report a male aged between 30 and 40 years old with LBP, who was submitted to a facet joint and ESI. The procedure evolved into a spinal infection of the psoas muscle. In addition, we present a brief literature review on psoas infections after spinal injection. Infection post facet joint lumbar block is very rare, with few publications in the literature. Early detection and the aggressive broad-spectrum antibiotic course must be initiated until adequate cultures are obtained and antibiotics prolong to at least four weeks.

## Introduction

Low back pain (LBP) is a common condition. It is estimated that 84% of adults will present LBP symptoms at some point in their lives [1]. However, some individuals continue to experience pain beyond the acute period of four weeks and may develop chronic back pain (persisting for more than 12 weeks). In rare cases, LBP is an indication of a serious medical condition, requiring further investigation [2].

The aetiology of LBP varies, but non-specific back pain represents up to 85% of cases [2]. This pain affects musculoskeletal structures like lumbar disks and facet joints, and are generally self-limiting, improving within a few weeks. Other causes include radiculopathy, vertebral fracture, spinal stenosis, spinal cord or cauda equine compression, metastatic cancer, and infection [2].

The treatment of non-specific LBP begins with non-pharmacological strategies such as superficial heat, massage, acupuncture or spinal manipulation, supplemented or not by non-steroidal anti-inflammatory drugs (NSAID), or skeletal muscle relaxants [3].

Because of the prevalence of LBP and other spine pain, non-surgical interventions have increased. The use of epidural steroid injections (ESI) or facet joint injections helps relieve pain in degenerative spine disorders or disk herniation. These therapies provide rapid pain relief, despite unsatisfactory long-term results [4].

Spine injection complications include bleeding with or without intravascular entry, dural puncture and spinal anaesthesia, neural trauma, spinal cord trauma, pneumothorax, radiation exposure, hematoma formation, and steroidal side-effects [5]. Infection is rarely described, although the American Society of Anaesthesiologists claim for 3% of infection after all blocks to pain, this complication after facet joint block is rarer. To the best of our knowledge, this is the first case report of psoas muscle infection following simple facet joint injection in the literature.

This paper describes a case of abscess in the psoas muscle, secondary to facet joint lumbar block, and describes diagnosis, evolution, and prognosis parameters.

## Case presentation

**Figure 1:**
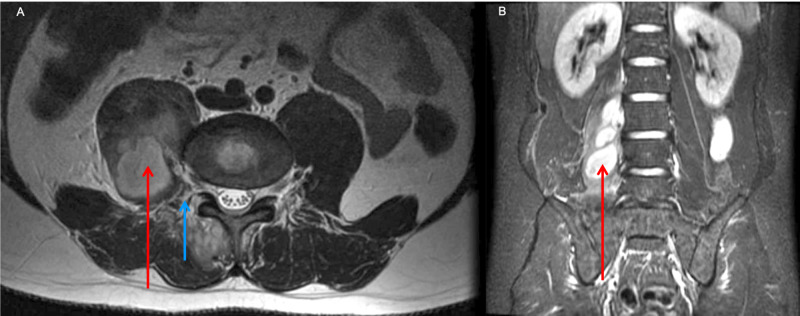
(A) Axial T2 MRI image showing the psoas muscle (red arrow) and facet joint lesion spreading to multifidus muscle (blue arrow). (B) Coronal T1 MRI post gadolinium showing extensive psoas muscle abscess (red arrow).

A 35-year-old male complaining of chronic LBP was submitted to an L2 to S1 bilateral facet joint and epidural injection (methylprednisolone plus lidocaine 1%), to improve pain symptoms. After ten days, the patient developed fever, malaise, right thigh pain, and antalgic hip flexion with no sign of meningeal or radicular insult. Laboratory evaluations revealed a haemoglobin count of 14.6 g/dl, and a white blood cell count of 21.8 k/cumm, with a neutrophilic predominance and 2% rod cells, C-reactive protein levels were at 122.8 mg/L, and an erythrocyte sedimentation rate (ESR) was recorded at 42 mm (60 min). Magnetic resonance imaging (MRI) of the lumbar spine indicated a coalescing abscess in the right psoas muscle, spreading from the facet joint (Figure 1).

**Figure 2:**
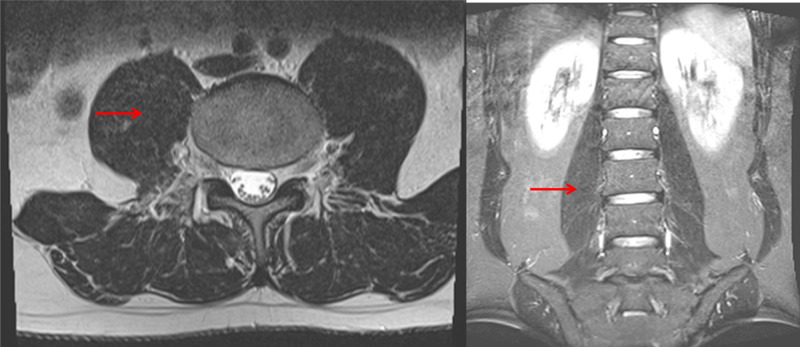
Three months follow-up MRI showing no signs of infection (red arrows): (A) axial plane and (B) coronal plane.

The patient was submitted to abscess puncture under fluoroscopy, with partial drainage. Samples were culture positive for Staphylococcus aureus and sensitive to oxacillin, ciprofloxacin, moxifloxacin, vancomycin, teicoplanin, and tigecycline. The patient was empirically treated with vancomycin and ceftriaxone for seven days, and after culture review, the patient completed an additional oxacillin course over seven additional days. The patient was discharged with oral ciprofloxacin for another 14 days treatment (total = 28 days). The patient recovered with complete regression of the infection at follow-up of one and three months (Fig. 2). After a month, the white blood cell count was 7.9 k/cumm with a lymphocytic predominance and no immature cells; C-reactive protein was 1.9 mg/L and the ESR was 20 mm (60 min).

## Discussion

Chronic LBP has a prevalence of 4.2% in individuals aged between 24 and 39 years old, and 19.6% in those aged between 20 and 59 [6]. In most cases, chronic LBP is investigated by appropriate physician evaluation and associated imaging tests when necessary [6].

Like other synovial joints in the human body, the lumbar facet joints represent a potential pain generator in patients with chronic LBP [7]. The prevalence has been reported ranging from 8% to 42% [7], the rates increase in conjunction with age in the populations studied. The term “facet syndrome” was coined by Ghormely in 1933; it is defined as lumbosacral pain, with or without sciatica, which was likely to occur after a sudden rotatory strain; he suggested that facet hypertrophy can lead to nerve root impingement and LBP [8]. More recently, most authors prefer to correlate clinical history, physical examination, radiological exams, and block tests. The hallmarks of facet pain include LBP without a radicular referral that is worse with standing, worse with lumbar extension and axial rotation, and improved with sitting or lumbar flexion. MRI is used to assess the soft tissue anatomy, and computed tomography (CT) is the imaging modality of choice to assess the details of bone and joint anatomy. With regard to the facet joints specifically, MRI can show facet effusions, facet cysts, and facet arthrosis, but arthrosis is far better defined by CT [9].

Unrelenting pain at rest and progressive neurological deficits should be evaluated with caution, especially for underlying cancer or infection. Imaging studies and a blood workup are mandatory in these patients, but other red flags include: pain maintenance despite treatment over 2-4 weeks, writhing or colicky pain associated with visceral functions, cancer diagnosis, fever or immunosuppressed status, high risk of fracture, associated malaise, fatigue or weight loss, bowel or bladder dysfunction, severe morning stiffness as the primary complaint, and incapacity to ambulate or care for oneself [10].

After initial improvement, the patient developed symptoms of alarm: fever, malaise, and changes in neurologic exams, turning detailed investigation mandatory.

Although techniques to perform facet joint injection may differ between studies, fluoroscopy is preferred, followed by ultrasound, CT, and MRI guidance which may be used for needle placement [11]. We used a solution of methylprednisolone acetate (40 mg/ml) and 1% lidocaine (equal volume), to inject approximately 1 ml into each facet joint. Other options include bupivacaine, ropivacaine, betamethasone, and triamcinolone [12].

Pyogenic arthritis of the lumbar facet joint is an extremely rare disorder, with an incidence rate of approximately 0.2% in reported spinal infections [13]. The literature presents infection cases post epidural injection for LBP in case report articles and small series [14-16]. Much more common are spondylodiscitis after facet joint steroid injection [17] and septic arthritis [18]. To the best of our knowledge, this is the first published case of psoas infection secondary to simple facet joint block.

Most of the time, spine infections are caused by S. aureus, especially in spinal epidural abscesses (30-80%). Other frequent pathogens include the coagulase-negative Staphylococci and Streptococci, and Escherichia coli which is also involved in spondylitis/spondylodiscitis and paraspinal abscesses. Fungal infections are rarely found, but those that occur predominantly include Aspergillus spp., Candida spp., and Cryptococcus neoformans. Unfortunately, in 33% of cases, no organisms are identified [19]. However, in this case study, S. aureus sensitive to oxacillin was identified and treated.

There are no guidelines or consensus articles regarding the use of antibiotic prophylaxis treatments for lumbar block procedures; therefore, there are no formal indications as it is rarely performed. Similarly, our patient did not receive any antibiotics prior to the first procedure. Treatments for spine infections must begin with empirical antibiotic administration until adequate culture results are available. Current protocols require ceftriaxone plus vancomycin until culturing dictates otherwise. Importantly, in our patients, cultures were rapidly made available and antibiotic therapy was adapted to S. aureus sensitive to oxacillin and ciprofloxacin [20]. Since no spondylodiscitis was associated with the case, a four-week treatment course was sufficient to eradicate the infection.

## Conclusions

Infections post facet joint lumbar block are very rare. Early detection and aggressive broad-spectrum antibiotic administration must be initiated, until adequate cultures are obtained. Selected antibiotics must be administered for at least four weeks.
